# Evidence for Active Maintenance of Inverted Repeat Structures Identified by a Comparative Genomic Approach

**DOI:** 10.1371/journal.pone.0000262

**Published:** 2007-02-28

**Authors:** Guoyan Zhao, Kuan Y. Chang, Katherine Varley, Gary D. Stormo

**Affiliations:** 1 Department of Genetics, Washington University School of Medicine, St. Louis, Missouri, United States of America; 2 Department of Pathology and Immunology, Washington University School of Medicine, St. Louis, Missouri, United States of America; Institute for Genomic Research, United States of America

## Abstract

Inverted repeats have been found to occur in both prokaryotic and eukaryotic genomes. Usually they are short and some have important functions in various biological processes. However, long inverted repeats are rare and can cause genome instability. Analyses of *C. elegans* genome identified long, nearly-perfect inverted repeat sequences involving both divergently and convergently oriented homologous gene pairs and complete intergenic sequences. Comparisons with the orthologous regions from the genomes of *C. briggsae* and *C. remanei* show that the inverted repeat structures are often far more conserved than the sequences. This observation implies that there is an active mechanism for maintaining the inverted repeat nature of the sequences.

## Introduction

An inverted repeat, or biological palindrome, consists of two arms of similar DNA sequences that occur adjacent to each other (perhaps containing a short, non-palindromic spacer between the arms) but on opposite strands and in opposite orientation. The consequence of these inverted repeats is that they can form hairpin or cruciform structures through intramolecular base pairing. Inverted repeats have been shown to play crucial roles in DNA replication [1], transcriptional regulation in various organisms from N4 bacteriophage to human [2]–[5], as well as translational control [6]. However inverted repeats are also one of the sources of genome instability and are known to cause different types of genomic rearrangements in a wide variety of organisms [7]–[9]. In human, inverted repeats are associated with several human diseases [10].

One type of genomic instability associated with inverted repeats is gene conversion, which is nonreciprocal transfer of genetic information. Whether gene conversion occurs is determined by how regions of heteroduplex DNA are resolved. Cruciform structure branch migration gives rise to regions of heteroduplex DNA. In the heteroduplex DNA region, if one strand (the acceptor) uses the other strand (the donor) as the template to repair mismatches, base changes occur only on the acceptor strand which results in a gene conversion event. Recently, it was proposed that gene conversion has maintained the structure and function of key genes in the non-recombinant region in the human Y chromosome [11].

In this report, we describe an interesting genomic structure of intergenic regions of *C. elegans*, *C. briggsae* and *C. remanei*. We found examples of intergenic regions between paralogous gene pairs that are inverted repeats and where the same genomic structure exists in all three species suggesting a common ancestry of the inverted repeat structures. Paralogous gene pairs and intra-palindromic (arm-to-arm) sequences exhibit unusually high sequence identity, sometimes 100% identity. However, orthologous gene pairs and orthologous palindrome arms are less conserved. These results suggest that some mechanism is functioning in all three species to maintain the inverted repeat structure which raises the possibility that the inverted repeat structure rather than the sequence plays a critical function.

## Results

### Conserved structure in *C. elegans*, *C. briggsae* and *C. remanei*

Many *C. elegans* intergenic regions have long inverted repeat sequences, but in the following we focus on a few examples with clear orthologous regions in *C. briggsae* and *C. remanei*. In each example a gene duplication event must have preceded the divergence of the species because the inverted repeat appears in each species. The orientation of the genes, both divergent and convergent, requires that the duplication event created an initial inverted repeat structure, rather than a direct repeat. But while the orthologous sequences have diverged considerably between species, the paralogous intergenic regions within species are often highly conserved.

**Figure 1:**
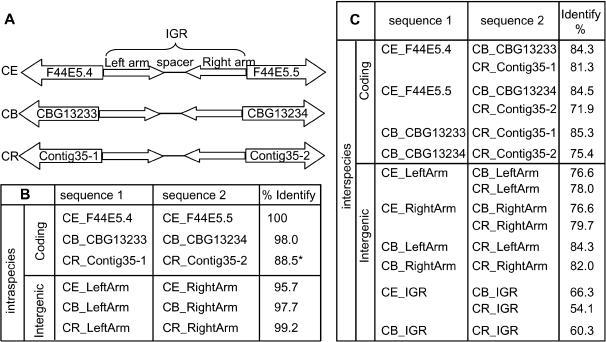
An example of divergently oriented inverted repeat. A. Schematic representation of the inverted repeat structure of the *C. elegans* F44E5.4 - F44E5.5 genomic region, CBG13233 - CBG13234 genomic region in *C. briggsae* and Contig35.Fgenesh-Celegans.59.final (Contig35-1) - Contig35.eannot.383.final.final (Contig35-2) region in *C. remanei*. B. Sequence comparison was carried out between sequence 1 and sequence 2 for each row. Sequence identity within each species (intraspecies) in a global alignment (Needleman-Wunsch) is shown in the last column of the table. C. Sequence identity between species (interspecies) is shown in the last column of the table. Sequence identity within a species is much higher than the sequence identity between species. CE: *C. elegans*; CB: *C. briggsae*; CR: *C. remanei*; IGR: intergenic region. * indicates that sequence identity could be higher than 88.5% because Contig35-2 has a stretch of Ns (10) which is estimated length of the sequencing gap.

Figure 1 shows one example of divergently oriented gene pairs. *C. elegans* genes F44E5.4 and F44E5.5 are paralogs that are divergently oriented with 100% DNA sequence identity. The intergenic sequence between F44E5.4 and F44E5.5 is 446 bp long with a 160 bp arm on each side and a 126 bp spacer. The sequence identity between the two arms is 95.7%. Similarly, *C. briggsae* genes CBG13233 and CBG13234 and *C. remanei* genes Contig35.Fgenesh_Celegans.59.final and Contig35.eannot.383.final.final, orthologs of F44E5.4 and F44E5.5, are divergently oriented with 98% and at least 88.5% DNA sequence identity, respectively. (Because the *C. remanei* genome is not finished yet, the *C. remanei* gene Contig35.eannot.383.final.final has a stretch of N, so the sequence identity could be higher than 88.5%.) The intra-palindromic arms of *C. briggsae* and *C. remanei* exhibit 97.7% and 99.2% sequence identity respectively. The existence of this palindromic structure in all three species suggests that a gene duplication event occurred before the separation of the three *Caenorhabditis* lineages. But sequence conservation is much higher between the paralogous palindromic arms within a species than between the orthologous sequences across species. In the example shown in figure 1, the palindromic arms of the inverted repeat structure have sequence identity greater than 95% within each species. However, the sequence identity of the palindromic arms between species is lower than 80% and the sequence identity of the entire intergenic regions are only 66.3% between *C. elegans* and *C. briggsae* and 54.2% between *C. elegans* and *C. remanei*. Therefore, it is the inverted repeat structure rather than the sequence that is conserved among all three species.

**Figure 2:**
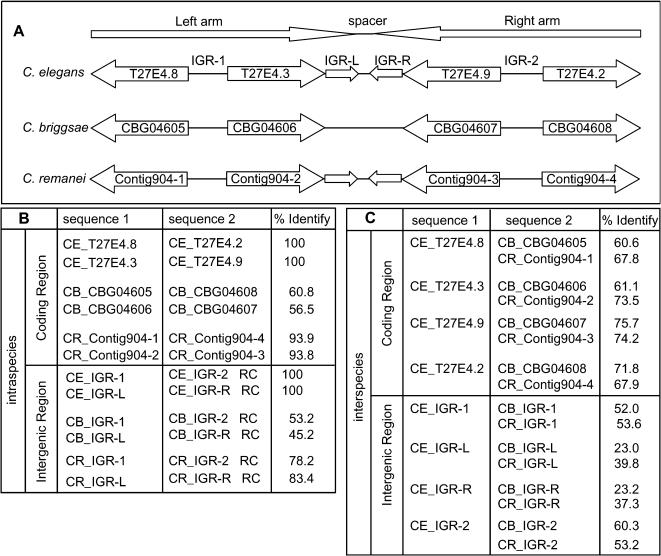
An example of convergently oriented inverted repeat. A. Schematic representation of the inverted repeat structure of *C. elegans* T27E4.3 - T27E4.9 genomic region as well as *C. briggsae* and *C. remanei* orthologous genomic region. In *C. elegans*, the region of T27E4.8, IGR-1, T27E4.3 and IGR-L is a perfect mirror image of the region of T27E4.2, IGR-2, T27E4.9 and IGR-R. In *C. remanei*, Contig904-snap.9.final (Contig904-1), Contig904-eannot.018.final.final (Contig904-2), Contig904.eannot.1017.final.final (Contig904-3) and Contig904.snap.4.final (Contig904-4) have similar inverted repeat structure. In *C. briggsae*, CBG04605, CBG04606, CBG04607 and CBG04608 are arranged in the same orientation but don't have inverted repeat structure. B. Sequence comparison was carried out between sequence 1 and sequence 2 for each row. Sequence identity within each species (intraspecies) in a global alignment (Needleman-Wunsch) is shown in the last column of the table. C. Sequence identity between species (interspecies) is shown in the last column of the table. Sequence identity within a species is much higher than the sequence identity between species for *C. elegans* and *C. remanei*. CE: *C. elegans*; CB: *C. briggsae*; CR: *C. remanei*; IGR: intergenic region.

Figure 2 gives one example of convergently oriented gene pairs. *C. elegans* HSP 16 gene locus consists of 4 genes that are arranged as a palindromic structure. The region of T27E4.8, IGR-1 (IGR: intergenic region), T27E4.3 and IGR-L is duplicated to generate an inverted repeat structure. The intergenic sequence between T27E4.3 and T27E4.9 is 662 bp long with a 124 bp arm on each side and a 414 bp spacer. The inverted repeat structure is perfectly maintained since duplication and the sequence identity between the palindromic arms is 100% both in the coding region and in the intergenic region. Because of the high sequence identity, it was proposed that the duplication event may have occurred recently or, alternatively, gene conversion may have maintained identity of the two gene pairs [12]. Our analyses suggest that this duplication event is an ancient one because a similar genomic structure also exists in *C. remanei*. *C. remanei* genes Contig904-snap9.final (Contig904-1), Contig904.eannot.018.final.final (Contig904-2), Contig904.eannot.1017.final.final (Contig904-3) and Contig904-snap4.final (Contig904-4) are arranged in the same orientations as their orthologs in *C. elegans* with 93.8% DNA sequence identity between Contig904-2, and Contig904-3 and 93.9% between Contig904-1 and Contig904-4. The palindromic arms of the intergenic sequence between Contig904-2 and Contig904-3 exhibit 83.4% sequence identity. Similarly, the sequence identity is much lower between orthologous gene pairs and between orthologous palindrome arms (Figure 2). In *C. briggsae*, genes CBG04605, CBG04606, CBG04607 and CBG04608 are arranged in the same orientations as their orthologs in *C. elegans* and *C. remanei*. However, sequence identity between the paralogous gene pairs as well as between the intergenic sequences are much lower (less than 61%). These data imply that *C. briggsae* inherited the same genomic structure generated by the same duplication event, but the inverted repeat structure was allowed to degenerate in *C. briggsae*. These results suggest that these inverted repeat structures are of ancient origin and are maintained in *C. elegans* and *C. remanei* but lost in *C. briggsae*.

### Duplication and evolution of paralogous gene pairs

We observed that divergently oriented paralogous gene pairs tend to duplicate as an entity and this duplication is still ongoing after speciation.

**Figure 3:**
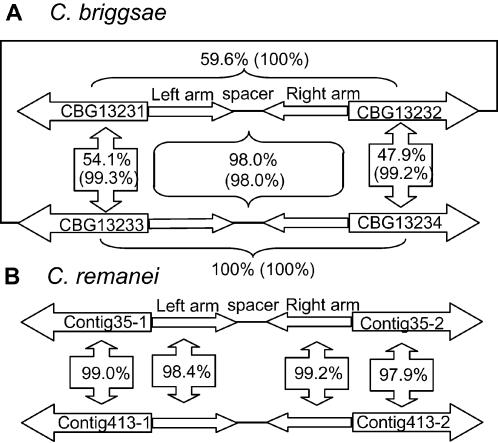
Duplication and evolution of inverted repeat orthologous gene pairs. A. Duplication of inverted repeat gene pair in *C. briggsae*. The CBG13233 - CBG13234 genomic region, which is orthologous to the *C. elegans* F44E5.4 - F44E5.5 genomic region, is duplicated as tandem repeats in *C. briggsae*. Number shows sequence identity between two sequences in a global alignment. Number in parenthesis shows sequence identity between two sequences in a local alignment. CBG13231 and CBG13233 are 99.3% identical for the first 297 nt. CBG13232 and CBG13234 are 99.2% identical for the first 764 nt. CBG13231 and CBG13232 are 100% identical for the first 297 nt. The intergenic region between CBG13231 and CBG13232 are 98% identical to the intergenic region between CBG13233 and CBG13234. B. Duplication of inverted repeat gene pair in *C. remanei*. The Contig35.Fgenesh-Celegans.59.final (Contig35-1) - Contig.eannot.388.final.final (Contig35-2) region, which is orthologous to the *C. elegans* F44E5.4 - F44E5.5 genomic region, is duplicated in *C. remanei*. Currently, it is not clearly whether the duplicated regions are in the same chromosome or not. Sequence identities between duplicated genes as well as between duplicated intergenic sequences are shown in the boxes.

Figure 3 shows that the orthologous region of F44E5.4 and F44E5.5 (in Figure 1) is duplicated in both *C. briggsae* and *C. remanei*. In *C. briggsae*, the region is duplicated as direct repeats (panel A). CBG13231 and CBG13233 are only 54.1% identical and CBG13232 and CBG13234 are only 47.9% identical in global alignment, suggesting that these genes have diverged considerably since duplication. However, CBG13231 and CBG13233 are 99.3% identical for the first 297 nt and CBG13232 and CBG13234 are 99.2% identical for the first 764 nt (numbers in parenthesis). Furthermore, the intergenic region between CBG13231 and CBG13232 is 98% identical to the intergenic region between CBG13233 and CBG13234. Sequence identity between CBG13231 and CBG13232 is only 59.6% globally but 100% identical for the first 297 nt. These results suggest that the inverted repeat structure between divergently oriented CBG13231 and CBG13232 is preserved extending to the coding region although the C-terminals of the genes have diverged considerably. Therefore, there must be some mechanism that maintained the inverted repeat structure but allowed the rest of the sequences to evolve differently. In *C. remanei*, the duplicated regions are in two different contigs. The intergenic regions only have 63.6% sequence identity as a whole. However, the left and right palindromic arms of the intergenic regions are 98.4% and 99.2% identical, respectively (Figure 3, panel B). Therefore, the spacer has evolved much faster than the coding region as well as the palindromic arms. Since *C. briggsae* and *C. remanei* are the most closely related species [13], it is not clear whether *C. elegans* lost the duplicated pair or that *C. briggsae* and *C. remanei* gained it in the branch leading to the separation of *C. briggsae* and *C. remanei*.

**Figure 4:**
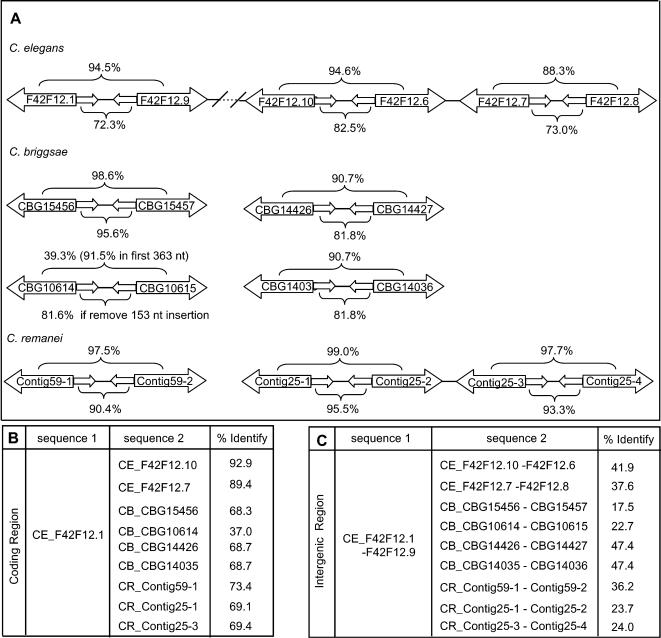
Duplication and evolution of inverted repeat gene pairs. A. Schematic representation of the genomic structure of the F42F12.1-F42F12.9 gene pairs as well as two *C. elegans* paralogous gene pairs, four *C. briggsae* orthologous gene pairs and three *C. remanei* orthologuos gene pairs. Numbers above each gene pair indicate the sequence identities between the two coding genes in a global alignment. Numbers below each gene pair indicate the sequence identifies between two arms of the inverted repeat intergenic region. Number in parenthesis shows sequence identity between the two sequences in a local alignment. B. Sequence identify between coding sequences. C. Sequence identity between intergenic sequences. For each row, sequence comparison was performed between sequence 1 and sequence 2 and sequence identity between these two sequences is shown in the last column. Contig59-1: Contig59.Fgenesh_Celegans.40.final; Contig59-2: Contig59.eannot.1190.final.final; Contig25-1: Contig25.Fgenesh_Celegans.92.final; Contig25-2: Contig.1332.final.final; Contig25-3: Contig25.Fgenesh_Celegans.94.final; Contig25-4: Contig.1333.final.final. CE: *C. elegans*; CB: *C. briggsae*; CR: *C. remanei*.

The divergently oriented paralogous gene pair of F42F12.1 and F42F12.9 and their paralogs and orthologs were duplicated in a more complex way (Figure 4). In *C. elegans*, three paralogous pairs exist on chromosome X: F42F12.1-F42F12.9, F42F12.10-F42F12.6, F42F12.7-F42F12.8 (panel A). F42F12.10-F42F12.6 and F42F12.7-F42F12.8 are adjacent to each other. F42F12.1-F42F12.9 and F42F12.10-F42F12.6 are separated by 12 kb genomic DNA with 5 genes. All three gene pairs have inverted repeat structures and sequence identities between coding gene pairs and between palindromic arms are shown in Figure 4. The *C. remanei* genome has three gene pairs that are homologous to the *C. elegans* genes with two pairs in the same contig. All three gene pairs have inverted repeat structures (panel A). The *C. briggsae* genome, however, has four gene pairs that are homologous to the *C. elegans* genes (panel A). Two interesting things are worth noting in *C. briggase*. First, gene pair of CBG10614-CBG10615 as well as their intergenic region has diverged considerably. CBG10614 and CBG10615 are 91.5% identical in the first 363 nt but CBG10614 (875 nt) is considerably longer than CBG10615 (363 nt). The inverted repeat structure in the intergenic region is disrupted by a 153 nt insertion. If the 153 nt insertion is removed, the palindromic structure is obvious with arms sharing 81.6% sequence identity. Second, CBG14426-CBG14427 and CBG14035-CBG14036 seem to be recent duplication because they are 100% identical in both coding regions and in intergenic sequences. Therefore, three out of four gene pairs maintained their inverted repeat structure although the sequences between orthologous gene pairs have diverged considerably (Figure 4, panel B, C). Because all four gene pairs are in different contigs, currently we do not know whether these duplications occurred before or after speciation. Completely finished genomic sequences of *C. briggsae* and *C. remanei* may help to answer this question.

## Discussion

In this study, we report our finding of highly conserved intergenic inverted repeat structures in less conserved intergenic sequences. Although inverted repeats have been the focus of several studies [14]–[17], this is the first time that inverted repeat structures involving paralogous gene pairs have been described. The presence of divergently/convergently oriented paralogs flanking the intergenic inverted repeat suggests that the inverted repeat was introduced during an intra-strand gene duplication in the common ancestor of the nematodes. Since *C. elegans* and *C. briggsase* were estimated to have diverged about 100 million years ago [18], it is not surprising that the intergenic sequences have diverged considerably. Conservation of the inverted repeat structure rather than the sequence in the three *Caenorhabiditis* species implies there is either a mechanism of symmetric mutation or that there is selective pressure retaining mutations that occur in the sequence which preserve the inverted repeat.

One mechanism to maintain the high conservation of inverted repeats is gene conversion. The analysis of the human Y chromosome revealed that several gene duplication events have occurred involving large inverted repeat sequences including coding regions. It was proposed that it is the palindromic arm to arm gene conversion that drives the paired arms to evolve in concert which results in the highly identical paired arms [11]. Gene conversion events have been described previously in *C. elegans* [17]. Perhaps a similar mechanism is maintaining the conserved inverted repeats in *C. elegans*, *C. briggsae* and *C. remanei*. The inverted repeat structure could be lost if free evolution is allowed. For example, the inverted repeat of CBG13231 and CBG13232 has only been partially preserved while the inverted repeat between CBG04606 and CBG04607 is completely lost in *C briggsae*.

However, long inverted repeats have been shown to have a profound effect on genome stability. In *E. Coli*, replicons with long inverted repeats (>150 bp) can not be propagated and are deleted at extremely high rates [19]. In yeast, a perfect palindrome, formed by two 1.0-kb inverted repeats, increased intra- and interchromosomal recombination in the adjacent region 2,400-fold and 17,000-fold, respectively and is also deleted at high frequency [20]. In mammals, inverted repeats are extremely unstable and undergo both homologous recombination and non-homologous deletion at high frequency [7], [21]. Inversion of the inverted repeat brought about by a homologous recombination will not stabilize the locus. The locus is stabilized only after the formation of central asymmetry by deletion [7], [21]. Although such studies have not been carried out in *C. elegans*, we would expect the *C. elegans* genome to have similar properties, based on the conservation between yeast and mammals. Genome instability is positively correlated with the size of inverted repeats, the identity between the stem arms and is negatively correlated with the size of intervening spacers [20]. In our study, the inverted repeat structure is very long (2207 bp of palindromic arms for the F44E5.4 locus) with a relatively short spacer (126 bp) and very high sequence identity (99.7%). This locus should be highly recombinagenic. However, they have been stably transmitted for about 100 million years. In addition, long inverted repeats with high sequence identity are very rare in the *C. elegans* genome (less than 0.7%) [15]. Therefore, the evidence suggests that the conserved inverted repeat structure in the intergenic sequence is due to selection for some function that requires the secondary structure allowed by an inverted repeat.

Conservation of DNA structure is observed in non-coding RNA genes where symmetric mutations are selected to preserve the intra-strand nucleotide base paring but not the overall sequence of the orthologs [22]. Since the sequence identity of the inverted repeats is low between species the function under selection as the species diverged must be associated with the structure. Inverted repeats have the potential to form cruciform structures *in vivo* [23]. Studies have shown that some cruciforms are critically involved in gene transcriptional regulation [23], [24]. Cruciforms may act as target sites for activator and repressor proteins and serve as a novel mechanism that controls cell-specific promoter activity [23]. In this study, the genes flanking the inverted repeats were always paralogs of each other. Perhaps the cruciform structure formed by the inverted repeat controls transcriptional regulation of the paralogs. Since some regulatory DNA binding proteins recognize the cruciform structure rather than the sequence [25] this could explain why the sequence identity of intergenic regions between orthologs is low, but the inverted repeat structure is conserved. A similar role was proposed for human inverted repeats in controlling sex-specific gene expression during germ-cell development or meiosis [26].

Currently, the genomes of many organisms have been sequenced. However, fully understanding of how information is stored in the genomes remains a big challenge. The novel genomic structures reported here suggests that there may be many more examples to be discovered and comparative genomics is a great tool for uncovering regions under unusual selection. It would be interesting to see whether similar structures are also present in other organisms and what is the biological function of this structure.

## Materials and Methods

### Identification of Inverted Repeats

We retrieved all *C. elegans* intergenic sequences and annotation from WormBase (http://www.wormbase.org/) and used a Needleman-Wunsch global alignment algorithm [27] to align an intergenic sequence against its reverse complement. This report is intended to identify specific intergenic regions that have significant repeats rather than to give a comprehensive list of gene pairs that have this genomic structure. Therefore, we use a stringent cutoff of 50% sequence identity to ensure that the intergenic region had a significant inverted repeat above the background of the *C. elegans* genome (Figure S1 shows the distribution of percent identity to the reverse complement for all *C. elegans* intergenic regions.).

### Identification of homologous gene pairs

We first identified all *C. elegans* gene pairs that have inverted repeats above the cutoff. We then identified all the gene pairs that are homologous to a given *C. elegans* gene pair in the genome of *C. elegans*, *C. briggsae* and *C. remanei*. To identify homologous gene pairs in the genome of *C. elegans* and *C. briggsae*, we used InParanoid Ortholog Groups information downloaded from http://inparanoid.cgb.ki.se/ [28]. In this study, the term ‘inparalogs’ indicate paralogs that arose through a gene duplication event after speciation, while ‘outparalogs’ arise following a gene duplication preceding speciation. We use this information as a guide to identify all the gene pairs that are homologous to a given *C. elegans* gene pair. We then analyzed the genomic regions of those genes for genomic structure and conservation. *C. briggsae* sequence and annotation were obtained from WormBase (http://www.wormbase.org/).

To identify the *C. remanei* orthologs for each of the genes flanking the inverted repeat in *C. elegans*, we used NCBI stand alone BLAST on the *C. remanei* proteome. We then determined if the orthologs in *C. remanei* were adjacent in the *C. remanei* genome and matched the gene orientation in *C. elegans*. Because we expect the gene pairs to be products of duplication, we did not require them to be mutual best BLAST hits. *C. remanei* sequence and annotation were produced by the Genome Sequencing Center at Washington University School of Medicine in St. Louis and can be obtained from http://genome.wustl.edu/pub/organism/Invertebrates/Caenorhabditis_remanei/.

## Supporting Information

Figure S1 The distribution of the percent identity of C. elegans intergenic sequences. X axis is the length of the intergenic sequence and the Y axis is the percent identity between intergenic sequence and its reverse complement. The red horizontal line represents cutoff.
(5.22 MB EPS)
